# 3D-printed collagen/silk fibroin/secretome derived from bFGF-pretreated HUCMSCs scaffolds enhanced therapeutic ability in canines traumatic brain injury model

**DOI:** 10.3389/fbioe.2022.995099

**Published:** 2022-08-24

**Authors:** Xiaoyin Liu, Guijun Zhang, Pan Wei, Lifang Hao, Lin Zhong, Kunhon Zhong, Chang Liu, Peng Liu, Qingbo Feng, Shan Wang, Jianyong Zhang, Rui Tian, Liangxue Zhou

**Affiliations:** ^1^ Department of Neurosurgery, West China Hospital, West China Medical School, Sichuan University, Chengdu, China; ^2^ Department of Neurosurgery, The First People’s Hospital of Long Quan yi District, Chengdu, China; ^3^ Department of Radiology, Liao Cheng The Third People’s Hospital, Liaocheng, China; ^4^ The First Affiliated Hospital of Chengdu Medical College, Chengdu, China; ^5^ State Key Laboratory of Biotherapy, West China Hospital, West China Medical School, Sichuan University, Chengdu, China; ^6^ Department of Liver Surgery and Liver Transplantation, State Key Laboratory of Biotherapy and Cancer Center, West China Hospital, Sichuan University, Chengdu, China; ^7^ Department of General Surgery, The Affiliated Hospital of Guizhou Medical University, Guiyang, China

**Keywords:** traumatic brain injury, secretome, human umbilical cord blood mesenchymal stem cells, bFGF, collagen, silk fibroin

## Abstract

The regeneration of brain tissue poses a great challenge because of the limited self-regenerative capabilities of neurons after traumatic brain injury (TBI). For this purpose, 3D-printed collagen/silk fibroin/secretome derived from human umbilical cord blood mesenchymal stem cells (HUCMSCs) pretreated with bFGF scaffolds (3D-CS-bFGF-ST) at a low temperature were prepared in this study. From an *in vitro* perspective, 3D-CS-bFGF-ST showed good biodegradation, appropriate mechanical properties, and good biocompatibility. In regard to vivo, during the tissue remodelling processes of TBI, the regeneration of brain tissues was obviously faster in the 3D-CS-bFGF-ST group than in the other two groups (3D-printed collagen/silk fibroin/secretome derived from human umbilical cord blood mesenchymal stem cells (3D-CS-ST) group and TBI group) by motor assay, histological analysis, and immunofluorescence assay. Satisfactory regeneration was achieved in the two 3D-printed scaffold-based groups at 6 months postsurgery, while the 3D-CS-bFGF-ST group showed a better outcome than the 3D-CS-ST group.

## Graphical Abstract

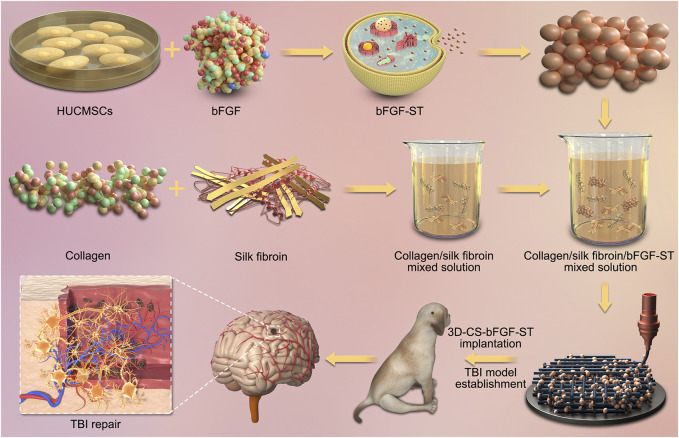

## Introduction

Considering that a growing number of the population suffers from traumatic brain injury (TBI), the incidence rate of TBI in children is 3–7%, and 1,568,346 adult patients have been diagnosed with TBI during the past eight years (Maas et al., 2017). TBI is, in accordance with the US Centers for Disease Control and Prevention, defined as a brain’s normal function disruption, which is caused by bumps, blows, jolts, or penetrating head injuries (Taylor et al., 2017). Patients with TBI may suffer cognitive, emotional, and behavioral inability in their remaining lifetime (Li et al., 2021). TBI is a major cause of morbidity and mortality and attracts a wide range of public concerns. Over the last few decades, it has led to a steep rise in therapeutic strategies in TBI populations by many scholars. Current treatment strategies for TBI are prioritized for the reduction of intracranial pressure and prevention of its subsequent complications (Alexis et al., 2015). Unfortunately, the application of surgery and drugs does not result in satisfactory neuroprotection and a favorable prognosis (Nichol et al., 2015).

Stem cell therapy, in recent years, has been generally accepted to be a promising and encouraging regenerative medical treatment in basic research fields about ischemic or hemorrhagic stroke, Alzheimer’s disease, and TBI(Wakai et al., 2016; Jahanbazi Jahan-Abad et al., 2018; Zhang et al., 2018; Huang et al., 2019; Bonsack et al., 2020; Han et al., 2020; Kawabori et al., 2020; Bae et al., 2021; Li and Sundström, 2022; Zhang et al., 2022). Mesenchymal stem cells (MSCs) can differentiate into various cell lineages under certain conditions: adipocytes, chondrocytes, glia, neurons, and osteoblasts. Considering its self-renewal, regenerative ability, and neuroprotective effect, MSC transplantation has been suggested as a therapeutic management strategy in animal models and clinical studies for structural and functional recovery (Tondreau et al., 2004). However, mounting evidence supports that stem cells confer a neurological benefit attributed to the corresponding secretome (ST), which exhibits similarly beneficial functions (Han et al., 2015; de Rivero Vaccari et al., 2016). It is composed of soluble factors and membrane vesicles. Soluble factors are transforming growth factors (Beer et al., 2017).

Of note, human umbilical cord blood mesenchymal stem cells (HUCMSCs) were reported to have advantages over other types of MSCs without limitations of immunological side effects or tumorigenesis as well as an aging-like nature and with easy accessibility and effective neurological protection (Bongso and Fong, 2013; Sharma et al., 2014; Pu et al., 2017; Hao et al., 2018; Sun et al., 2018). It is currently believed that ST derived from HUCMSCs harbored an ability of tissue regeneration, reducing the possibility of immune rejection. ST could be regarded as a potentially strong nanomedicine for TBI treatment, and we hypothesized that HUCMSC-ST could compensate for the damaged neurons and neurotrophy. However, recent studies revealed that unconjugated ST was unable to be sustained in the injured area during the recovery process (Kankala et al., 2017; Metzger et al., 2017). To solve the problem of retention and stability at different storage levels, our study advocated that scaffold-based 3D printing technology could overlay HUCMSC-ST (Jiang et al., 2021).

Increasing evidence has confirmed that several biomaterials may resemble the microenvironment of the extracellular matrix, and one of the major types of tissue engineering is the use of 3-dimensional (D) scaffolds. Among these scaffolds, collagen (C), chitosan, and silk fibroin (SF), as the most valuable candidate materials for biomedical applications, are popular for cell culture, growth, and differentiation (Zhang et al., 2017). In addition, in a previous study, we found that collagen and silk fibroin (CS) had a favorable effect on a canine model of TBI(Jiang et al., 2021). Moreover, 3D bioprinting was beneficial to a more comforting placement and distribution of living cells, thereby allowing for the construction of bioactively complex microenvironments.

Basic fibroblast growth factor (bFGF) is a small peptide of a large family that regulates cell proliferation, differentiation, and migration (Xie et al., 2013). In addition, it has been reported that the production of angiogenic factors, such as bFGF and vWF, enhances the innate proangiogenic ability of MSCs(Wei et al., 2008).

With advances in tissue engineering techniques, a combination of materials and stem cells has provided promising neurogenesis results. Based on this technique, bFGF was added and supposed to increase the quality and quantity rate of cells *in vitro* and at the injury site. On the basis of the 3D-printed scaffold, we developed a novel tactic to promote the therapeutic effects of secretome derived from HUCMSCs pretreated with bFGF (bFGF-ST). We incorporated bFGF-ST into collagen/silk fibroin mixed solution to form 3D-printed collagen/silk fibroin/secretome derived from HUCMSCs pretreated with bFGF scaffolds (3D-CS-bFGF-ST), which could release bFGF-ST into the surroundings in a sustained manner.

Thus, combining these technologies, a 3D-printed platform incorporating bFGF-ST at a low temperature was described in this study. We aimed to evaluate the potential effects of 3D-CS-bFGF-ST on tissue regeneration and motor function recovery after TBI in canine models.

## Materials and methods

### Isolation and characterization of HUCMSCs and bFGF-ST

The umbilical cords of healthy pregnant women who gave birth naturally or by cesarean section were washed, cut and digested with collagenase and 0.25% trypsin (Liu et al., 2020). HUCMSCs were incubated in a saturated humidity of 37°C and 5% CO_2_ at an inoculation density of 1 × 10^6^/cm^2^. The culture medium was changed after 24 h at the first time point and then once every 3 days. The suspension cells were passaged at a ratio of 1:1. To determine the biological characteristics of HUCMSCs. We used the immunological phenotypes CD90 (1:300, Abcam, Cambridge, UK) and CD 105 (1:400, Abcam, Cambridge, UK) based on immunofluorescence staining.

For the isolation of bFGF-ST, briefly, the primary MSCs were seeded in T75 (75-cm^2^) culture flasks and maintained in DMEM with 10% fetal bovine serum for 24 h. Then, DMEM containing bFGF (PeproTech) (100 ng/ml bFGF in complete medium) was used to replace the conditioned medium. After HUCMSCs were pretreated with bFGF for 24 h, serum-free, low-glucose DMEM was used to replace the conditioned medium. Conditioned media were harvested at 24 h. The conditioned media was centrifuged once at 500×g for 10 min and then twice at 800×g for 15 min. The bFGF-ST secreted by 1×10^7^ cells was concentrated into 20 μL. Two types of secretome were collected in our study: normal HUCMSCs secretome without pretreatment (ST), the HUCMSCs secretome pretreated with bFGF (bFGF-ST). A bicinchoninic acid (BCA) protein assay kit (Beyotime, China) was performed to measure the protein concentrations of ST and bFGF-ST.

### Preparation of 3D-CS-bFGF-ST

Collagen was isolated from fresh bovine tendons, and purification of collagen was carried out in accordance with an established method described previously (Liu et al., 2019; Sun et al., 2019). To sufficiently improve purities, the tendons were crushed and soaked in a buffer solution (0.05 mol/L) for 24 h. The precipitate, acquired by centrifugation at 2000 rpm, was added to acetic acid solution containing pepsin to obtain the supernatant. Next, 3.5 mol/L NaCl solution was added to the supernatant for salting out. Consequently, the collagen gel was obtained by dialysis at 4°C for 5 days.

Silk fibroin was produced following the established method (Yuhui et al., 2011; Xu et al., 2016; Jiang et al., 2020). One hundred grams of silk (Jiaxing, Zhejiang, China) was prepared and boiled in a 0.5% NaHCO_3_ (Solarbio Science & Technology Co., Ltd., Beijing, China) solution for dehydration. Subsequently, the dried silk was added to a ternary solution of CaCl_2_·CH_3_CH_2_OH·H_2_O (Solarbio Science & Technology Co., Ltd., Beijing, China) with a molar ratio of 1:2:8 at 80°C. Through subsequent filtration, dialysis, and concentration, the SF solution was obtained and stored at 4°C.

To fabricate scaffolds, collagen and silk fibroin were evenly mixed at a mass ratio (collagen:silk fibroin = 1:10) for 3D printing preparation. Before 3D printing, 0.1 g collagen/silk fibroin mixed solution was soaked in 20 μL bFGF-ST solution (200 μg) at 4°C for 24 h, followed by stirring at 4°C for 12 h, in order to fully and uniformly mix the collagen/silk fibroin mixture and bFGF-ST together. For low-temperature 3D printing technology, a 3D bioprinter (Regenovo Biotechnology Co., Ltd., Hangzhou, China) was designed to create constructs at −20°C. Computer aided design (CAD) to form a multihole support template. Modelling software (Solidworks software, Dassault Systèmes, Vélizy, France) was performed to use the 3D printing model for this study, followed by converting the predesigned STL 3D model into G code. The printing parameters were as follows: normal feed speed at 10 mm/s, ordinary extrusion speed at 2 mm/s, and first layer print height at 0.7 mm/s. Print layer thickness at 0.32 mm/s, print layer = 7 layers, print needle diameter at 260 µM, bottom plate molding temperature at −20°C. After 3D printing, the 3D solid model was freeze-dried and then cut into cylindrical scaffolds (2 mm diameter, 2 mm height) by using molds. The six types of scaffolds used in this study are as follows: 3D-printed collagen scaffolds (3D-C) group, 3D-printed collagen/secretome derived from HUCMSCs scaffolds (3D-C-ST) group, 3D-printed collagen/secretome derived from HUCMSCs pretreated with bFGF scaffolds (3D-C-bFGF-ST), 3D-printed collagen/silk fibroin (3D-CS), 3D-printed collagen/silk fibroin/secretome derived from HUCMSCs scaffolds (3D-CS-ST) and 3D-printed collagen/silk fibroin/secretome derived from HUCMSCs pretreated with bFGF scaffolds (3D-CS-bFGF-ST).

### *In vivo* degradation

Thirty Sprague–Dawley (SD) rats, 2 months old and weighing approximately 300 g, were used. The scaffold degradation *in vivo* was evaluated according to the difference in their weights with 5 different mass ratios (collagen:silk fibroin = 1:2, 1:6, 1:10, 1:14, 1:18). A small incision, 3 small at approximately 1 cm in length, was made on SD rat backs after anesthesia. The three sterilized scaffolds from one group were implanted in the lesions from one rat. The final weights were recorded at predetermined time intervals of 1–6 months. Then the scaffolds were placed in 2% sodium dodecyl surface (SDS) and the 2% SDS was replaced every 12 h. After 2 days, the scaffold was placed in ultrapure water. Through sterilization by DNase-RNase I digestion fluid (Solarbio Science & Technology Co., Ltd, China) overnight and shaking at a constant temperature of 37°C for 48 h, the decellularized scaffolds were placed in 30 ml of PBS and incubated at 37°C. Percent mass remaining = (decellularized scaffold mass/initial mass) × 100%.

### Characterization of the 3D-printed scaffold

Optical images and microscopic morphologies were obtained by using confocal laser scanning microscopy (CLSM, LSM 880, Zeiss), digital camera and stereomicroscope (SZX16, Olympus), respectively. The distribution and surface morphology of the scaffold were analysed by a typical HE staining procedure, which was reported previously. The physical characteristics of the scaffold, including the absorption ratio, porosity ratio, and mechanical strength, were calculated according to a previous method (Liu et al., 2021). A synchronous differentiated thermal analyser was used to analyse the phase transition temperatures of bFGF-ST, 3D-CS, and 3D-CS-bFGF-ST. The samples headed at an increase from 30 to 400°C. The scaffolds were evaluated by Braker X-ray diffraction. Its relevant parameters were as follows: tube voltage = 40 kV, tube current = 30 mA, target material was CuKα = 0.15406 nm, scanning speed = 5°C/min, step width = 0.02°, and diffraction angle range = 5–90°C. Cumulative secretome release from 3D-CC-ST was examined by using the bicinchoninic acid (BCA) reagent test kit (Beyotime) according to a previous study (Li et al., 2020; Guan et al., 2022).

### Assessment of scaffold cytocompatibility

To analyse the effect of the 3D-printed scaffold on cells *in vitro*, HUCMSCs at passage 3 were dissociated into single cells and plated at a concentration of 1×10^6^/ml into the scaffold, which was divided into two groups: 3D-CS-ST and 3D-CS-bFGF-ST. The proliferation of HUCMSCs on the two kinds of scaffolds was assessed by phase-contrast microscopy (Nikon, Tokyo, Japan), scanning electron microscopy (SEM) (Hitachi, Tokyo, Japan), hematoxylin and eosin (HE) staining, and MTT assay (Solarbio Science & Technology Co., Ltd) according to a previous study (Liu et al., 2021). After 7 days of coculture, through serial dehydration in ethanol, samples were dried in a CO_2_ critical point dryer and then sputter-coated with gold using SEM analysis for the morphology and growth of the cells. HE staining was used to evaluate the growth status of NSCs on 3D-CS-ST and 3D-CS-bFGF-ST.

NSCs were isolated from embryonic day 14 (E14) brains (Wakai et al., 2016). The dissociated cells were cultured in a mixed medium of DMEM/F12 (1:1) (Gibco. Unitad States) supplemented with N2 (Gibco), B27 (Gibco), 20 ng/ml bFGF and 20 ng/ml epidermal growth factor (EGF) (PeproTech). These cells were incubated in a humidified incubator at 37°C and 5% CO_2_. Immunofluorescence staining for Nestin, NF, GAP43 and NeuN was used to identify NSCs and assess the degree of differentiation of NSCs. NSCs were also seeded on two types of scaffolds (3D-CS-ST and 3D-CS-bFGF-ST) in a flat-bottomed 12-well plate at a density of 1×10^6^/ml. The cell adhesion rate was measured at 1, 12, 24, 36, 48, 60 and 72 h. Cells were treated with MTT solution, and the absorbance was measured on days 1, 3, 5, and 7 at 490 nm with a microplate reader.

### Canine TBI models and scaffold implantation

A total of 20 male canines, 1 year old and weighing 11–14 kg, were obtained. The established TBI model has been enunciated in the literature (Jiang et al., 2018). Following anesthesia, a preconditioned traumatic cortical motor injury occurred on the right side through a craniotomy. 3D-CS-bFGF-ST and 3D-CS-ST were implanted to fill up the lesions. According to the different treatment strategies, the animals were randomly assigned to 4 groups: the Sham group (Only the skull was opened without TBI, *n* = 5), the TBI group (TBI without scaffolds implantation, *n* = 5), the 3D-CS-ST group (3D-printed collagen/silk fibroin/secretome derived from HUCMSCs scaffolds were implanted into the TBI cavity, *n* = 5), and the 3D-CS-bFGF-ST group (3D-printed collagen/silk fibroin/secretome derived from HUCMSCs pretreated with bFGF scaffolds were implanted into the TBI cavity, *n* = 5). General anesthesia was performed on each canine to minimize the pain caused by the surgery.

### Evaluation of locomotor function

The neurological function assessment of all canines was performed by two independent authors at 1 day, 1 week, 2, 4, 8, 20, and 24 weeks after surgery (*n* = 5 for each group). Based on the modified GCS (mGCS) with a range from 3–18 (Platt et al., 2001), the Purdy scale with a range from 2–11 (Purdy et al., 1989), and the NDS score with a range from 0–500 (Castellá et al., 2005), they were used to comprehensively understand the behavioral level and locomotor function force measures.

### Histological analysis and immunofluorescence assay

Six months after the surgery, HE staining, Bidschowsky’s silver staining, Masson staining, Nissl staining, and Luxol Fast Blue (LFB) staining were used according to the manufacturer’s instructions to detect cerebral cortex repair in canine brain tissue (*n* = 5 for each group) (Jiang et al., 2020; Zhu et al., 2020; Jiang et al., 2021; Zhu et al., 2022). Nissl staining using toluidine blue staining was performed to observe the distribution of dead neurons. Briefly, paraffin sections (5 µM) were prepared, dehydrated with gradient alcohol staining with toluidine blue solution, and washed with distilled water. Glacial acetic acid staining was performed on the sections. LFB, the sections were dewaxed and placed in ethanol, and following stain-filtered LFB for 2–4 h at 60°C, they were placed in ethanol, distilled water, and lithium carbonate solutions in turn. After silver staining, first, the sections were dewaxed, dehydrated in ethanol, and then placed in silver nitrate solution at 37°C for 30 min in the dark. After washing in distilled water and placing in formaldehyde solution, the slices were dehydrated and dewed with xylene. Masson staining was performed to observe glial fiber growth. The paraffin sections were dewaxed and dehydrated in descending concentrations of ethanol and then washed in leak warm running top water for 3 min. After staining in hematoxylin and rinsing with distilled water, they were placed in aniline blue dye and glacial acetic acid solution after freshly filtered phosphomolybdic acid was added. Immunofluorescence staining was based on previous reports (*n* = 5 for each group) (Jiang et al., 2020, 2021). First, after dewaxing the paraffin slices, EDTA buffer was used for antigen repair of cerebral cortex sections. Slices were then incubated with brine serum albumin (BSA (Abcam)) for 30 min to send the serum. Second, slices were incubated in a wet box overnight at 4°C with the following primary antibodies: MBP: rat anti, 1:1,000, Abcam, Cambridge, UK; NEFM: rabbit anti, 1:1,000, Proteintech, Unitad States; Syn: rabbit anti, 1:1,000, Bioss, Beijing, China; MAP-2: chicken anti, 1:1,000, Abcam, Cambridge, UK; GAP-43: chicken anti, 1:1,000, Abcam, Cambridge, UK; PSD95: rabbit anti, 1:1,000, Abcam, Cambridge, UK; Tuj-1: rabbit anti, 1:1,000, Abcam, Cambridge, UK; vWF: rabbit anti, 1:1,000, Abcam, Cambridge, UK). Secondary antibodies were added after removal of the primary antibody. They were incubated in the dark for 50 min at room temperature. Then, DAPI staining was performed and incubated at room temperature for 10 min. The slices were sealed, and the images were observed and collected through a fluorescence microscope. TUNEL staining (Promega Corporation) was performed to assess the apoptosis rate of cells in the area of TBI at 6 months after TBI according to a previous study (Zhang et al., 2021) (*n* = 5 for each group).

### Enzyme-linked immunosorbent assay

ELISA (Wuhan Servicebio Technology Co., Ltd., China) was performed to analyse the relative levels of TNF-α, IL-6, and IL-10 in canine serum at the acute (1 week) and chronic stages (6 months) following the manufacturer’s instructions (*n* = 5 for each group). Briefly, the samples were plotted in 24-well plates and incubated with biotinylated antibodies and termination solution analysed by ELISA.

### Statistical analysis

Quantitative data in this study, from at least three biological replicates, were expressed as the mean ± standard deviation (SD). One-way ANOVA followed by connections was used to compare differences among multiple groups. Analysis was carried out with GraphPad 8.0 software. A *p* < 0.05 was considered statistically significant.

## Results

### Fabrication, mechanical property and degradation test of 3D-CS-bFGF-ST

**FIGURE 1:**
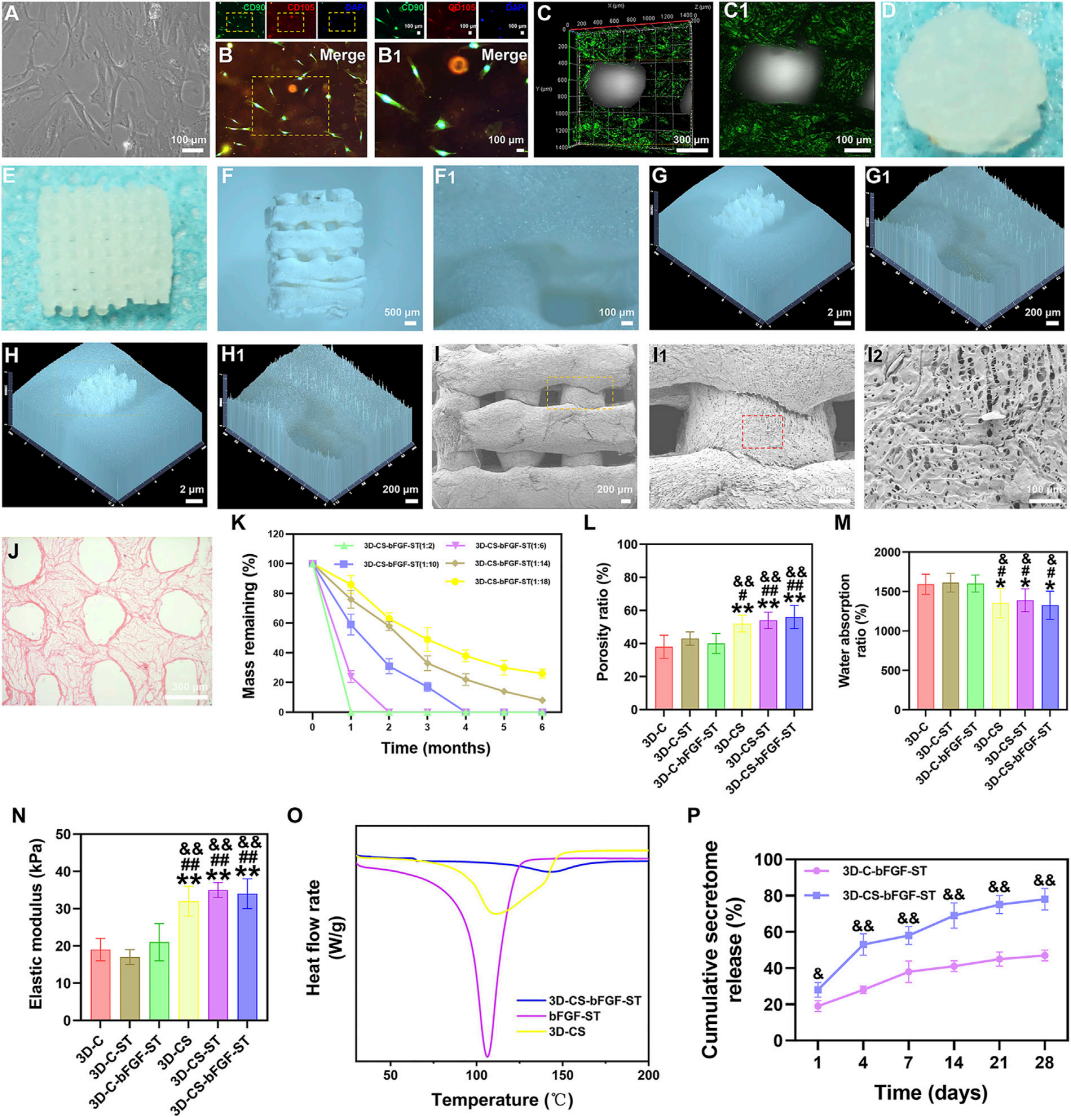
**(A)** HUCMSCs morphology under phase contrast microscopy. **(B–B1)** Expression of CD90 (green) and CD105 (red) in HUCMSCs under immunofluorescence. **(C–J)** Representative morphology of 3D-CS-bFGF-ST by using CLSM **(C–C1)**, digital camera **(D–E)** and stereomicroscope **(F–H1)**, SEM **(I–I2)**, and HE staining **(J)**. **(K)** The degradation test of 3D-CS-bFGF-ST. **(L–N)** Mechanical behavior of six kinds of scaffolds. The porosity ratio **(L)**, water absorption ratio **(M)**, and lastic modulus **(N)** of the six kinds of scaffolds. **(O)** DSC curves of 3D-CS, bFGF-ST and 3D-CS-bFGF-ST. **(P)** Cumulative secretome release of 3D-CS-bFGF-ST. ^*^ *p* < 0.05, ^**^ *p* < 0.01 vs. 3D-C. ^#^ *p* < 0.05, ^##^ *p* < 0.01 vs. 3D-C-ST. ^&^ *p* < 0.05, ^&&^ *p* < 0.01 vs. 3D-C-bFGF-ST.

Under a phase-contrast microscope, we observed a fibroblast similar to HUCMSCs (Figure 1A), and immunofluorescence staining identified HUCMSCs that strongly expressed CD90 and CD105 in our study (Figure 1B,B1). A porous construct with multiple layers in a tailored size was printed using a 3D bioprinter. The surface and micromorphology of 3D-CS-bFGF-ST were obtained by using CLSM, digital camera and stereomicroscope (SZX16, Olympus), SEM, and HE staining (Figures 1C–J).

The degradation of the 3D-CS-bFGF-ST-based performance with collagen and silk fibroin was tested (Figure 1K). At one month after transplantation, 3D-CS-bFGF-ST with a mass ratio of 1:2 were totally degraded (Figure 1K). Along with the decrease in the mass ratio of collagen/silk fibroin, the degradation rate of 3D-CS-bFGF-ST increased. Approximately 74% of 3D-CS-bFGF-ST with a mass ratio of 1:18 had been degraded at half a year after transplantation (Figure 1K). Approximately 69% of 3D-CS-bFGF-ST with a mass ratio of 1:10 was degraded from the time of transplantation to 2 months after transplantation, and approximately 17% of the mass was retained at 3 months after transplantation, which indicated that 3D-CS-bFGF-ST with a mass ratio of 1:10 was a suitable mass ratio among the different scaffolds (Figure 1K). Subsequent experiments in this study selected 3D-CS-bFGF-ST with a mass ratio of 1:10.

We focused on differences across the entire 3D-printed scaffold among the six groups in terms of the porosity ratio, the water absorption ratio, and the elastic modulus. Compared to 3D-C, 3D-C-ST and 3D-C-bFGF-ST, a significant increase in the porosity ratio of 3D-CS, 3D-CS-ST and 3D-CS-bFGF-ST was observed, leading to an improvement in the diffusion of nutrients as well as tissue formation (*p* < 0.05) (Figure 1L). The water absorption ratios of 3D-CS, 3D-CS-ST and 3D-CS-bFGF-ST were significantly lower than those of 3D-C, 3D-C-ST and 3D-C-bFGF-ST (*p* < 0.05) (Figure 1M). A significant increase was also observed in the elastic modulus of 3D-CS, 3D-CS-ST and 3D-CS-bFGF-ST compared with 3D-C, 3D-C-ST and 3D-C-bFGF-ST (*p* < 0.01) (Figure 1N). To further demonstrate the stability of ST, which was caused by temperature value. The Tm value of 3D-CS-bFGF-ST was significantly higher than that of bFGF-ST (Figure 1O). It should be noted that the higher the value temperature is, the better the stability. Therefore, the DSC results indicate that the combination of 3D-CS-bFGF-ST and bFGF-ST could increase the stability of bFGF-ST. We observed a significant increase in the cumulative secretome release of bFGF-ST in the 3D-CS-bFGF-ST group compared with the 3D-C-bFGF-ST group at each point (days 1, 4, 7, 14, 21, 28), indicating that the addition of silk fibroin could promote the fixation and absorption of ST to this scaffold (Figure 1P).

### Influence of 3D-CS-bFGF-ST on stem cells proliferation and differentiation

**FIGURE 2:**
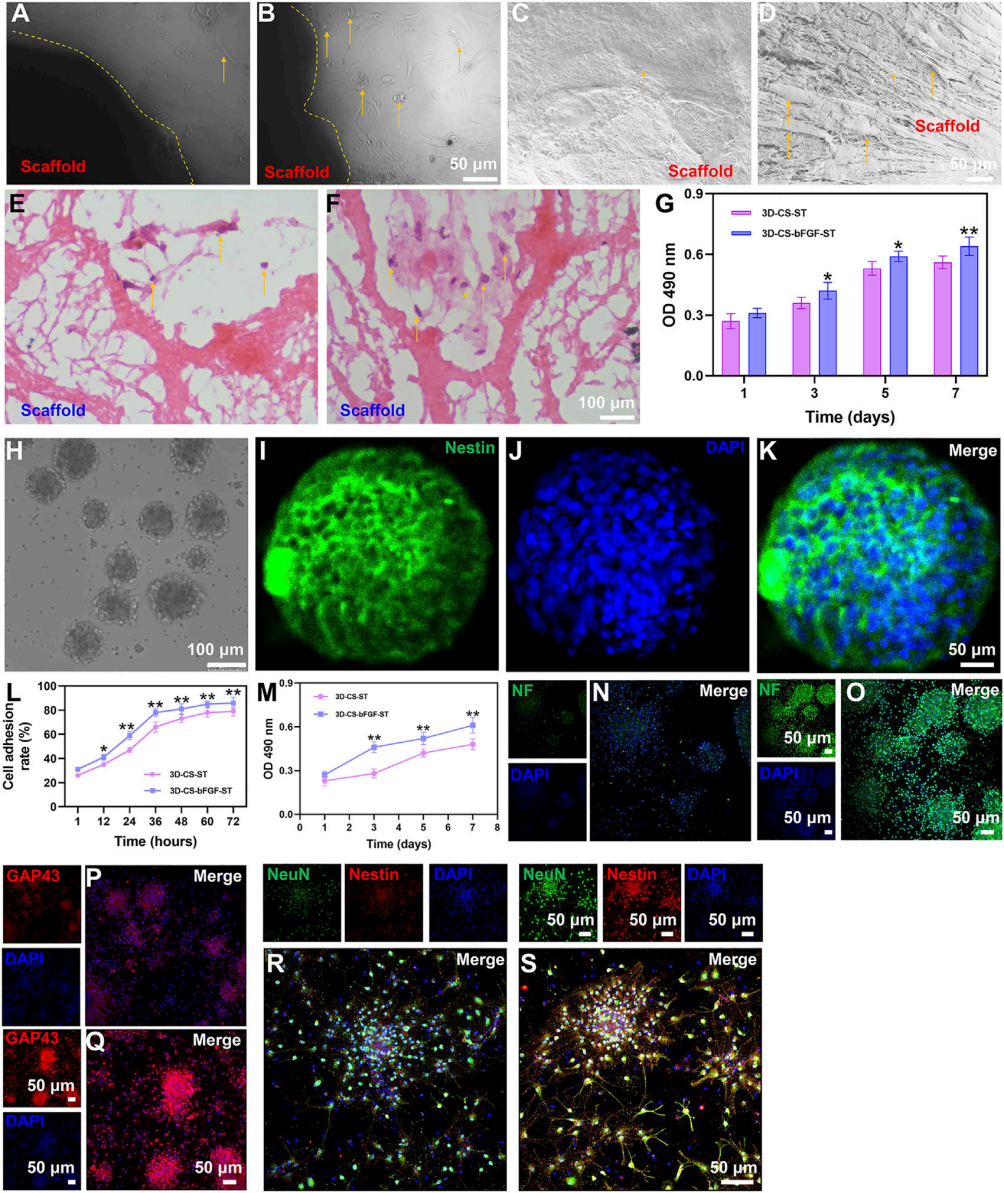
Typical morphology of cocultures of the two kinds of scaffolds (3D-CS-ST **(A,C,E)** or 3D-CS-bFGF-ST **(B,D,F)** and HUCMSCs under phase-contrast microscopy, SEM and HE staining. **(G)** MTT results of cocultures of the two kinds of scaffolds and HUCMSCs. **(H)** Typical morphology under an inverted fluorescence microscope. **(I–K)** Identification of NSCs by Nestin staining. **(L)** Cell adhesion rate of the 3D-CS-ST group and the 3D-CS-bFGF-ST group. **(M)** MTT results of cocultures of the two kinds of scaffolds and NSCs. (N–S) Expression of NF **(N–O)**, GAP43 **(P–Q)**, and NeuN **(R–S)** in NSCs after coculture. ^*^ *p* < 0.05, ^**^ *p* < 0.01 vs. 3D-CS-ST.

3D-CS-bFGF-ST and 3D-CS-ST were examined with phase-contrast microscopy, SEM and HE staining. The 3D-CS-based scaffolds were divided into 2 different groups and then cultured for 1, 3, 5, and 7 days. On the seventh day after coculture, the results of phase-contrast microscopy, SEM and HE staining indicated that the number of HUCMSCs in the 3D-CS-bFGF-ST group was significantly higher than that in the 3D-CS-ST group (Figures 2A–F). For the results of MTT, although the significant differences did not differ between the two groups (3D-CS-ST and 3D-CS-bFGF-ST) at day 1, a larger number of cells was seen in the 3D-CS-bFGF-ST group compared to the 3D-CS-ST group at days 3, 5, and 7 (*p* < 0.05) (Figure 2G). These results suggested that the addition of ST pretreated with bFGF to 3D-printed collagen/silk fibroin scaffolds could further increase cell proliferation.

NSCs in this study exhibited typical NSCs spheroid morphology under an inverted fluorescence microscope (Figure 2H). Identification of NSCs was performed by Nestin staining (Figures 2I–K). The typical morphology of immunofluorescent staining of NSCs after 7 days of coculture with both scaffolds was shown. Next, significant differences in NSCs adhesion and proliferation were also determined. MTT indicated that the cell adhesion rate increased over time (Figure 2L). A higher level of adhesion on 3D-CS-bFGF-ST was shown compared to the 3D-CS-ST group (Figure 2L). The absorption at 490 nm was in accordance with the NSCs viability of the 3D-CS-bFGF-ST group and the 3D-CS-ST group, and good cell growth was observed in both groups (Figure 2M). The proliferation of NSCs showed a dissimilar growth trend between these two scaffolds. The cell viability in the 3D-CS-bFGF-ST group remained at a relatively slow growth rate from day 3 to day 5 but greatly increased from day 1 to day 3. The cell viability in the 3D-CS-ST group grew slowly from day 1 to day 3 and from day 5 to day 7, but it grew at a high speed from day 3 to day 5. From the first day, we did not observe any significant difference in cell growth, while significant differences between these two scaffolds were observed on days 3, 5, and 7.3D-CS-bFGF-ST had a higher optical density (OD) value than 3D-CS-ST on days 3, 5, and 7 (*p* < 0.01) (Fig. 2M). Compared to the 3D-CS-ST group, the 3D-CS-bFGF-ST group showed stronger expression of NF, GAP43, and NeuN, which suggested that 3D-CS-bFGF-ST could promote axonal growth and facilitate the formation of mature neurons (Figure 2P–S).

### 3D-CS-bFGF-ST ameliorated functional scores after TBI

**FIGURE 3:**
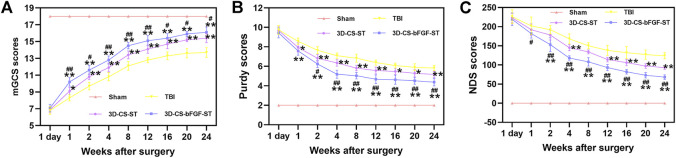
mGCS score **(A)**, Purdy score **(B)** and NDS score **(C)** of the four groups. ^*^ *p* < 0.05, ^**^ *p* < 0.01 vs. TBI. ^#^ *p* < 0.05, ^##^ *p* < 0.01 vs. 3D-CS-ST.

Neurological deficits post TBI were evaluated with regard to the mGCS score, Purdy score, and NDS score, which indicated that 3D-CS-bFGF-ST could significantly increase the neurological score in TBI in a time-dependent manner than in the 3D-CS-ST group and the TBI group, excluding 1 day after transplantation (Figures 3A–C). A significantly higher mGCS score was found in the 3D-CS-bFGF-ST group compared to the 3D-CS-ST group and the TBI group (*p* < 0.05) (Figure 3A). The NDS score was significantly higher in the 3D-CS-bFGF-ST group than in the other two groups at each point after 2 weeks (*p* < 0.05) (Figure 3B). Significantly higher NDS scores were found in the 3D-CS-bFGF-ST group than in the TBI group and the 3D-CS-ST group (*p* < 0.05) (Figure 3C). All of these results indicate that at the same time postoperation (from 4 to 24 weeks), canines treated with 3D-CS-bFGF-ST exhibited beneficial recovery.

### Implanting 3D-CS-bFGF-ST reduced the cavity area and glial scar and enhanced the formation of nerve fibers, myelin sheaths, and neurons in the TBI injury area

**FIGURE 4:**
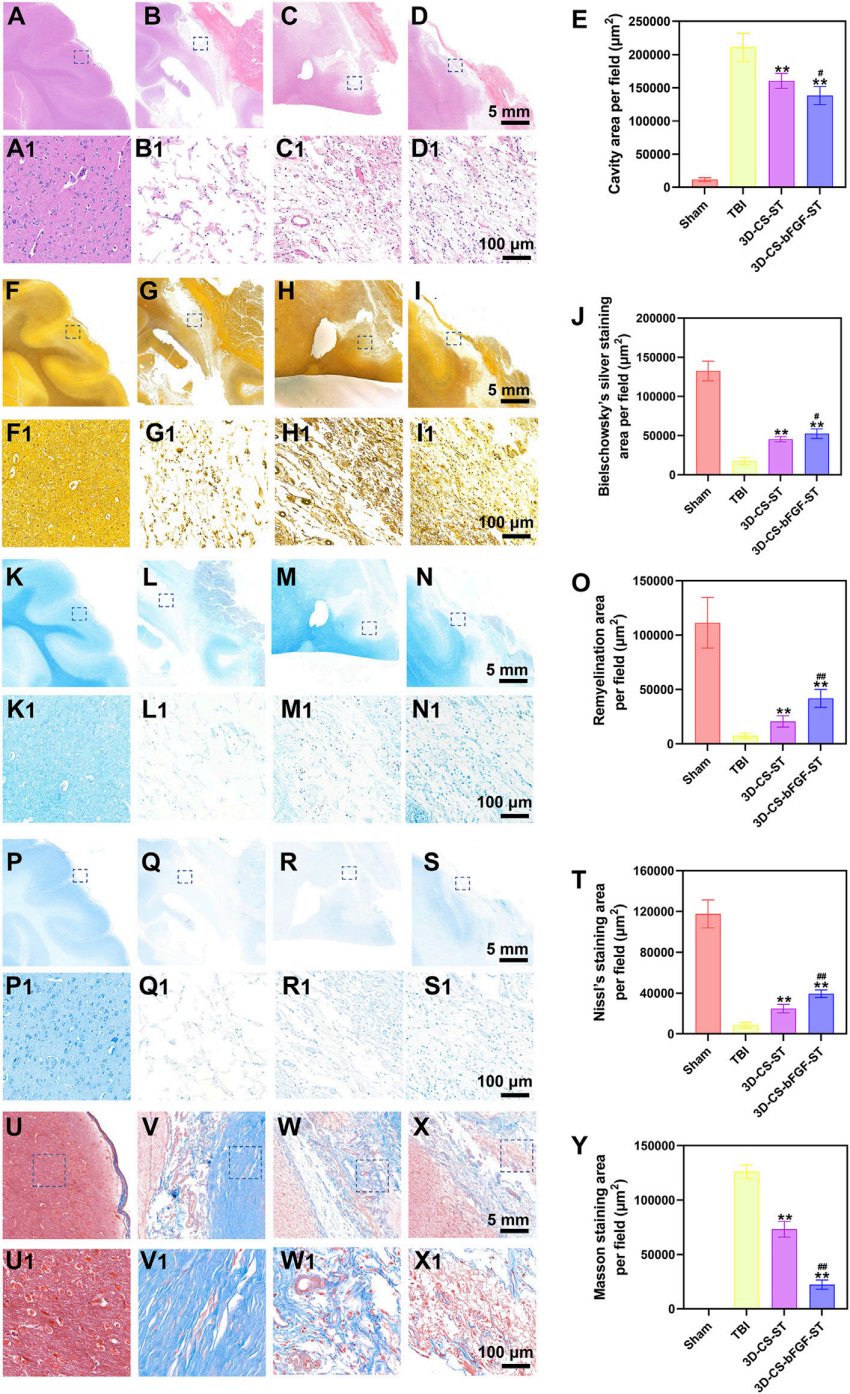
**(A-E)** Representative HE staining images **(A-D1)** in Sham group **(A** and **A1)**, TBI group **(B and B1)**, 3D-CS-ST group **(C and C1)**, and 3D-CS-bFGF-ST group **(D and D1)**, and their quantitative analysis **(E)**. **(F-J)** Representative Bielschowsky's silver staining images **(F-I1)** in Sham group **(F and F1)**, TBI group **(G and G1)**, 3D-CS-ST group **(H and H1)**, and 3D-CS-bFGF-ST group **(I and I1)**, and their quantitative analysis **(J)**. **(K-O)** Representative LFB staining images **(K-N1)** in Sham group **(K and K1)**, TBI group **(L and L1)**, 3D-CS-ST group **(M and M1)**, and 3D-CS-bFGF-ST group **(N and N1)**, and their quantitative analysis **(O)**. **(P-T)** Representative Nissl staining images **(P-S1)** in Sham group **(P and P1)**, TBI group **(Q and Q1)**, 3D-CS-ST group **(R and R1)**, and 3D-CS-bFGF-ST group **(S and S1)**, and their quantitative analysis **(T)**. **(U-Y)** Representative Masson staining images **(U-X1)** in Sham group **(U and U1)**, TBI group **(V and V1)**, 3D-CS-ST group **(W and W1)**, and 3D-CS-bFGF-ST group **(X and X1)**, and their quantitative analysis **(Y)**. **P < 0.01 vs TBI. #P < 0.05, ##P < 0.01 vs 3D-CS-ST.

Histological evaluation of injured tissue was performed at 6 months after transplantation. HE staining revealed a large cavity area with a disordered structure in the TBI group (Figure 4A–D1). In contrast, a smaller cavity with a well-disordered structure was detected in the 3D-CS-bFGF-ST group and the 3D-CS-ST group (Figure 4A–D1). Compared with implantation of 3D-CS-ST, implantation of 3D-CS-bFGF-ST could significantly reduce the TBI cavity area (Figures 4A–E). In regard to Bielschowsky’s silver staining, samples in the TBI-only group exhibited considerable vacuolar necrosis, without nerve fiber regeneration, while in the 3D-CS-bFGF-ST group and the 3D-CS-ST group, favorable synapse-like struck with regeneration of nerve fibers was displayed (Figures 4F–I). Larger Bielschowsky’s silver staining was observed in the 3D-CS-bFGF-ST group than in the 3D-CS-ST group (Figures 4F–J). Few blue myelin sheaths were found around the damaged area by LFB staining in the TBI group, while a larger number of blue-stained myelin sheaths were found in the 3D-CS-bFGF-ST group and the 3D-CS-ST group (Figure 4K–N1). The remyelination area in the 3D-CS-bFGF-ST group was larger than that in the 3D-CS-ST group (Figure 4K–O). Nissl staining, a method for assessing the diversity of neurons that survive, was used to show the overall morphology of the injured brain in the chronic phase of TBI. An abundant loss of neurons occurred in the TBI group, while implantation of 3D-CS-bFGF-ST and 3D-CS-ST filled the cavity and prohibited neuron loss (Figure 4P–S1). Compared with implantation of 3D-CS-ST, implantation of 3D-CS-BFGF-ST could further increase the area of Nissl staining (Figure 4P–T). Masson staining showed that compared with the TBI group, samples in the 3D-CS-bFGF-ST group and 3D-CS-ST group showed a significant decrease in glial fibers in the lesions (Figure 4U–X1). Notably, in regard to both treatment groups, the 3D-CS-bFGF-ST group displayed a more satisfactory outcome (Figure 4U–Y).

### Implanting 3D-CS-bFGF-ST accelerated the regeneration of nerve fibers, myelin sheaths and axons *in vivo* after TBI

**FIGURE 5:**
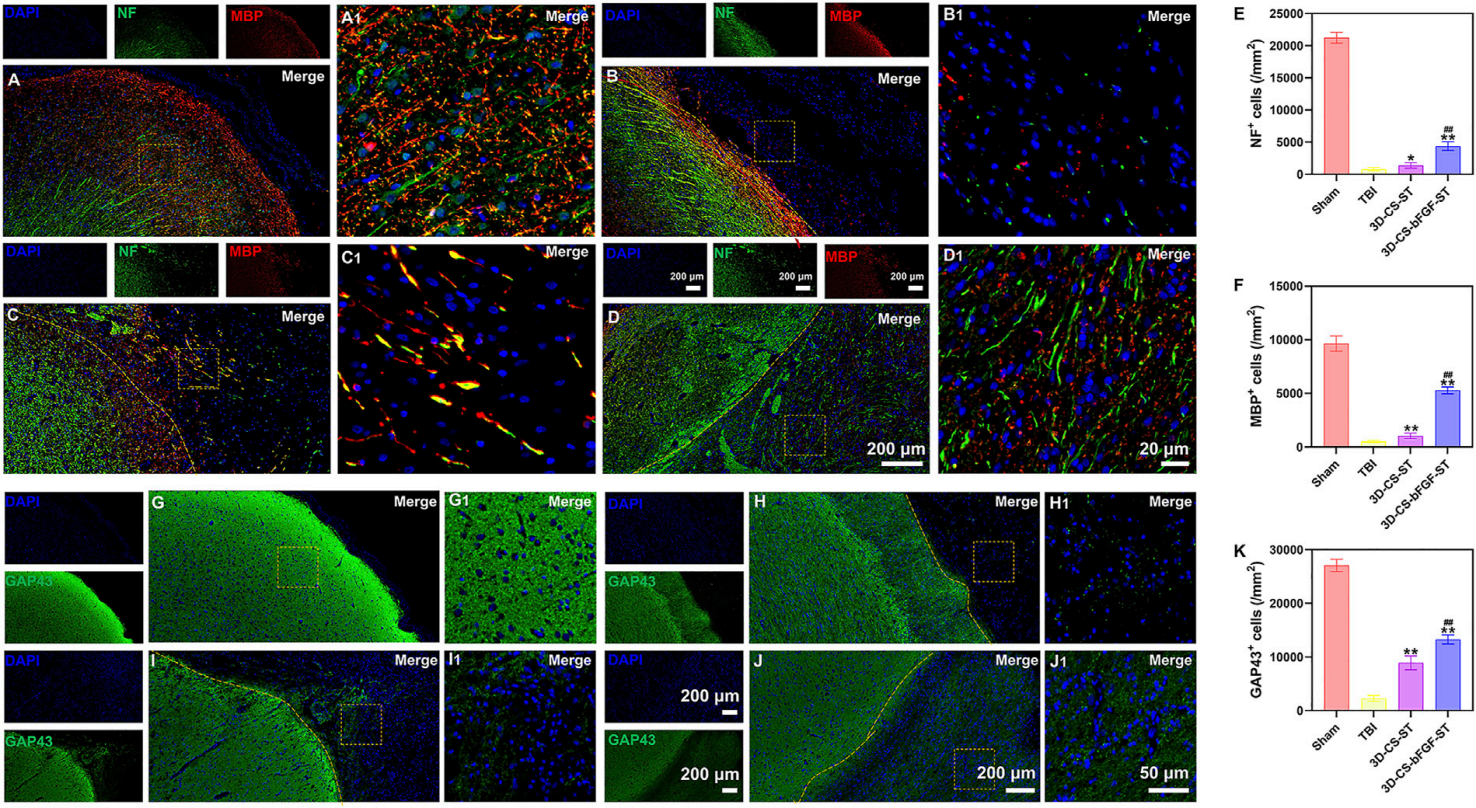
**(A-D1)** Typical picture of double-labelled immunofluorescence staining (NF (green) and MBP (red)) in Sham group **(A and A1)**, TBI group **(B and B1)**, 3D-CS-ST group **(C and C1)**, and 3D-CS-bFGF-ST group **(D and D1)**. **(E-F)** Quantitative analysis of NF and MBP. **(G-J1)** Typical immunofluorescence staining picture of GAP43 (green) in Sham group **(G and G1)**, TBI group **(H and H1)**, 3D-CS-ST group **(I and I1)**, and 3D-CS-bFGF-ST group **(J and J1)**. **(K)** Quantitative analysis of GAP43. *P < 0.05, **P < 0.01 vs TBI. ##P < 0.01 vs 3D-CS-ST.

Double-labelled immunofluorescence staining (NF and MBP) was performed to determine neuronal regeneration in the peri-injured tissue after TBI, and the number of immunofluorescence-positive cells was counted. The 3D-CS-bFGF-ST group displayed more NF- and MBP- positive cells than the other two groups (the TBI group and the 3D-CS-ST group), which supported that local transplantation could facilitate the regeneration of nerve fibers and myelin to some degree (Figures 5A–F). In addition, compared to the 3D-CS-ST group, the regeneration-promoting effects of 3D-CS-bFGF-ST were more obvious (Figures 5A–F). Similar results were also found for the expression of GAP43, which regulates synaptic plasticity and axonal regeneration, and this effect was most obvious in the 3D-CS-bFGF-ST group among the three injury groups (Figures 5G–K).

### Implanting 3D-CS-bFGF-ST improved neuron and synapse formation after TBI

**FIGURE 6:**
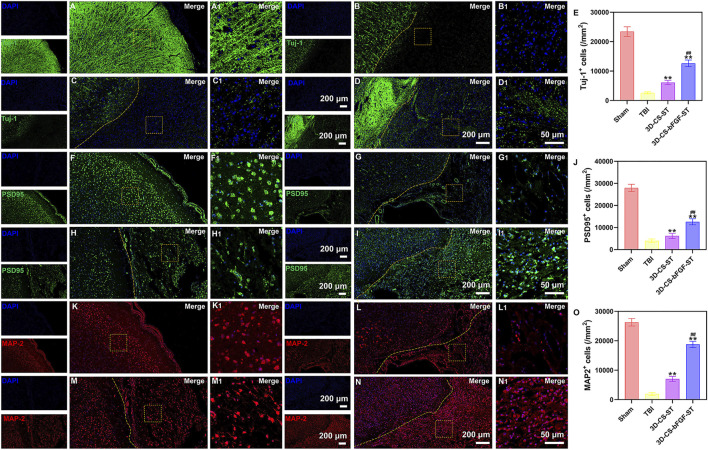
**(A-D1)** Typical immunofluorescence staining picture of Tuj-1 (green) in Sham group **(A and A1)**, TBI group **(B and B1)**, 3D-CS-ST group **(C and C1)**, and 3D-CS-bFGF-ST group **(D and D1)**. **(E)** Quantitative analysis of Tuj-1. **(F-I1)** Typical immunofluorescence staining picture of PSD95 (green) in Sham group **(F and F1)**, TBI group **(G and G1)**, 3D-CS-ST group **(H and H1)**, and 3D-CS-bFGF-ST group **(I and I1)**. **(J)** Quantitative analysis of Tuj-1. **(K-N)** Typical immunofluorescence staining picture of MAP-2 (red) in Sham group **(K and K1)**, TBI group **(L and L1)**, 3D-CS-ST group **(M and M1)**, and 3D-CS-bFGF-ST group **(N and N1)**. **(O)** Quantitative analysis of MAP-2. **P < 0.01 vs TBI. ##P < 0.01 vs 3D-CS-ST.

Compared with the TBI group and the 3D-CS-ST group, the expression of Tuj-1 (a marker of neuronal differentiation) in the 3D-CS-bFGF-ST group had significantly more positive cells per unit area (Figures 6A–E). The levels of PSD95, a marker for synapse formation, were significantly higher in cells in the 3D-CS-bFGF-ST group and the 3D-CS-ST group (Figures 6F–J). In contrast, few cells in the TBI group exhibited synaptic transmission (Figures 6F–J). More PSD95-positive cells were observed in the 3D-CS-bFGF-ST group than in the 3D-CS-ST group (Figures 6F–J). MAP-2 plays important roles in neurogenesis, including stabilizing microtubules, and determining and stabilizing dendritic shape during neuronal development. The number of MAP-2 positive cells in the 3D-CS-bFGF-ST group and the 3D-CS-ST group was significantly higher than that in the TBI group (Figure 6K–O). Treatment with 3D-CS-bFGF-ST conferred a better outcome than treatment with 3D-CS-ST (Figure 6K–O).

### Implanting 3D-CS-bFGF-ST could enhance angiogenesis, reduce cell apoptosis and modulate inflammatory levels after TBI

**FIGURE 7:**
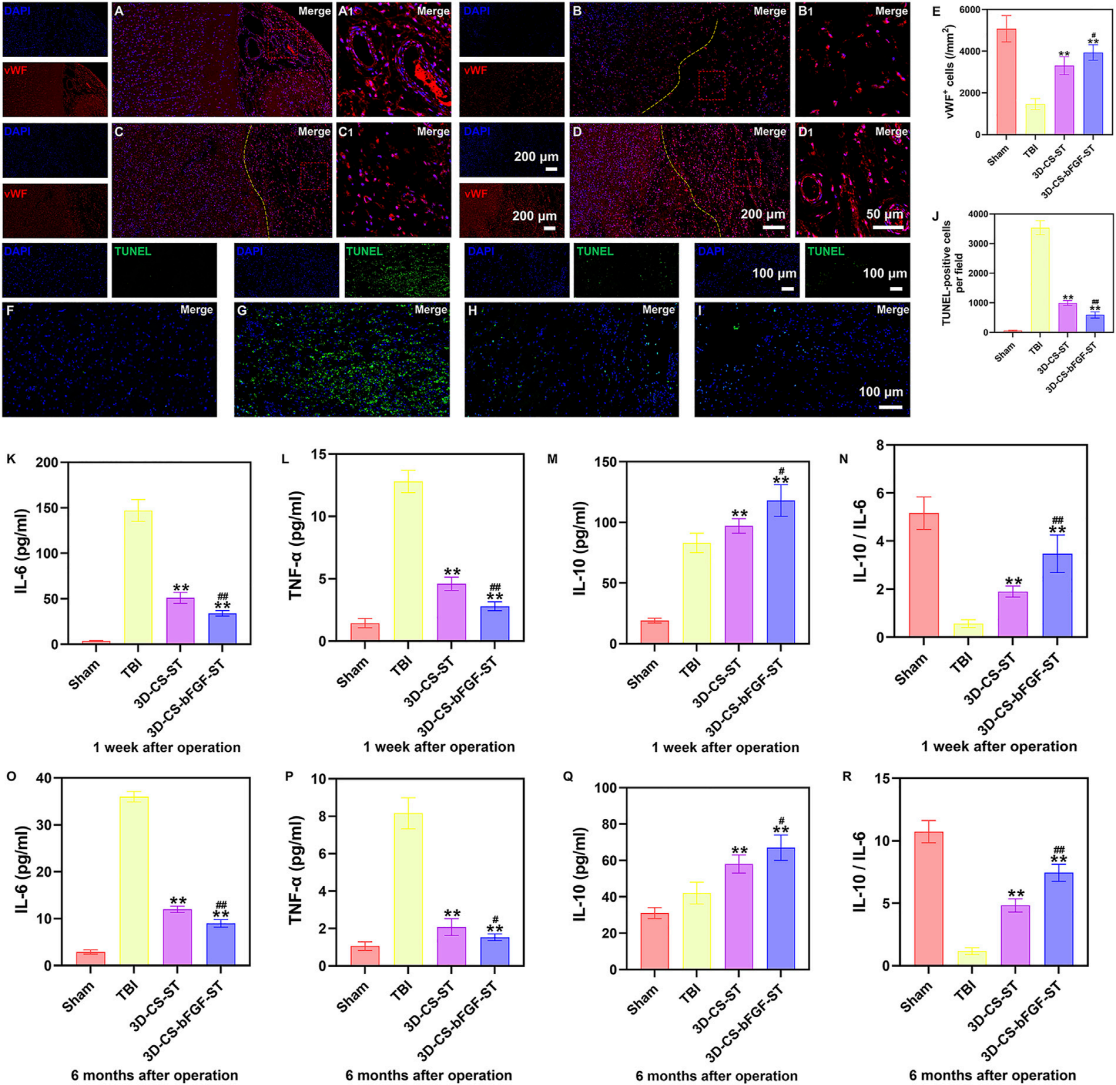
**(A-D1)** Typical immunofluorescence staining picture of vWF (red) in Sham group **(A and A1)**, TBI group **(B and B1)**, 3D-CS-ST group **(C and C1)**, and 3D-CS-bFGF-ST group **(D and D1)**. **(E)** Quantitative analysis of MAP-2. **(F-I)** Typical TUNEL (green) staining picture. **(J)** Quantitative analysis of TUNEL in Sham group **(F)**, TBI group **(G)**, 3D-CS-ST group **(H)**, and 3D-CS-bFGF-ST group **(I)**. **(K-N)** The expression of IL-6 **(K)**, TNF-α **(L)**, IL-10**(M)**, and IL-10/IL-6 **(N)** at 1 week after TBI. **(O-R)** The expression of IL-6 **(O)**, TNF-α **(P)**, IL-10 **(Q)**, and IL-10/IL-6 **(R)** at 6 months after TBI. **P < 0.01 vs TBI. #P < 0.05, ##P < 0.01 vs 3D-CS-ST.

In the 3D-CS-bFGF-ST and 3D-CS-ST groups, a larger number of vWF (a marker for vessel formation)-positive cells were captured in the perilesional area, especially in the 3D-CS-bFGF-ST group (Figures 7A–E). TUNEL staining was used to determine neuronal apoptosis in the preinjured brain. A large number of TUNEL-positive cells were found around the lesion site. Significant differences were detected between the TBI group and the 3D-CS-bFGF-ST and 3D-CS-ST groups, which indicated that treatment with 3D-CS-bFGF-ST and 3D-CS-ST could reduce cell apoptosis (Figures 7F–J). Fewer TUNEL-positive cells were found in the 3D-CS-bFGF-ST group than in the 3D-CS-ST group (Figures 7F–J). The expression of IL-6, TNF-α, and IL-10 was detected by ELISA at 1 week and 6 months after TBI. Lower IL-6 and TNF-α expression levels, higher IL-10 expression levels and higher IL-10 to IL-6 ratios were observed in the 3D-CS-bFGF-ST group than in the TBI group and 3D-CS-ST at both the chronic and acute stages, which indicated that implanting 3D-CS-bFGF-ST could modulate the level of inflammation after TBI (Figure 7K–R).

## Discussion

The brain shows a limited repair ability after injury due to the lack of neural regeneration and vascularization. Stem cells are usually isolated from embryonic cells in the blastocyst or from adult tissues, with the capability of self-renewal and differentiation toward a diversity of cell types (Clevers et al., 2014; Jayarajan and Milsom, 2020). It is commonly accessible to ST from stem cells as a priority in tissue regeneration. However, how modifications assist ST in some aspects of regeneration remains disputed. bFGF is capable of witnessing neurite outgrowth (Mie et al., 2014), and its stimulation of the ST may be an appropriate cue for tissue engineering. HUCMSC-derived ST incorporated in the scaffold by transplantation into canines has been examined, but no method has yet been developed to study 3D-CS with secretome derived from HUCMSCs pretreated with bFGF *in vitro* and *in vivo*. Thus, the current study using the 3D-printed scaffold incorporating HUCMSC-ST pretreated with bFGF supported the increased benefit of this complexes.

Neuro regeneration scaffolds have attracted significant attention for improving the virtuously desirable features. Currently, for example, the collagen or silk fibroin scaffold, a type of naturally biodegradable and biocompatible polymeter, has been used in a wide range of tissue engineering applications owing to its high affinity for adherent cells (Chlapanidas et al., 2013; Kundu et al., 2013; Liu et al., 2017). Collagen appeared as an alternative to the extracellular matrix due to its most abundant, firm, fibrous, and structural protein, but its application was limited due to its poor mechanical strength and thermostability (Liu et al., 2019). Silk fibroin is extensively used to produce composite scaffolds, mainly consisting of core filament protein, which can compensate for mechanical strength and extensibility/toughness by the crystalline heavy chains and amorphous light chains (Kundu et al., 2013; Koh et al., 2015). Therefore, collagen or silk fibroin blending served as a promising candidate for improving biological and physical characteristics because of its impressive mechanical properties and its excellent cell proliferation, differentiation and matrix deposition (Ghezzi et al., 2011). The scaffold fabrication could be attributed to the 3D printing technology, but it is still challenging in porosity, water absorption and elastic modulus, and the most importantly, the suitable behavior for even distribution for bFGF-ST loading. Hence, this study developed a novel strategy using ice-templated assembly and temperature gradient-guided thermally induced phase separation.

Moreover, we identified that bFGF pretreated ST in the scaffold and regulated neuronal induction at the injury site. In general, the degradation of the fabricated scaffolds was estimated by implanting the scaffolds *in vivo* with their remaining degradation products, and desirable degradation could be obtained by modifying the mass ratio of collagen/silk fibroin. 3D-CS-bFGF-ST exhibited good mechanical strength, which is an important factor affecting cell survival, neuronal differentiation, and axon formation. Studies on the regeneration potential effects of bFGF on the neuro-regeneration of HUCMSCs or ST derived from HUCMSCs have been carried out with tissue places or 3D environments in previous research. A satisfying porous structure was observed in 3D-CS-bFGF-ST, which indirectly affects the swelling ratio, indicating the transfer of waste and nutrition in and out of the scaffold (Chowdhury et al., 2018).

We compared the influence of ST and bFGF-ST in HUCMSCs or NSCs seeded on 3D-CS scaffolds. SEM and HE assays demonstrated that our scaffold had the capability for cell adhesion and proliferation. Figure 2 revealed that there was a significant difference in the cell viability of HUCMSC-ST based on the MTT test between the 3D-CS-ST group and the 3D-CS-bFGF-ST group. In the current study, significant differences in HUCMSCs and NSCs proliferation were observed between the 3D-CS-ST group and the 3D-CS-bFGF-ST group, suggesting the superiority of bFGF. In addition, to determine whether HUCMSC-ST pretreated with bFGF also have a profound influence on the differentiation of NSCs, the relative expression, including the expression of NF, GAP43 and NeuN, was determined after 7 days of coculture. The results of confocal microscopy revealed that 3D-CS-bFGF-ST had a high synergistic differentiation property and had the potential to provide a permanent microenvironment for cell−cell communication and NSCs spreading and differentiation.

Since the complexes had good biocompatibility *in vitro*, we utilized a canine-based TBI model to explore the change in functional recovery and neural regeneration. On the one hand, similar neuropathological changes and cerebrovascular abnormalities, such as neuronal loss, impaired neurogenesis, and cognitive decline, have been observed in both humans and canines (Giaccone et al., 1990; Uchida et al., 1990; Ellis et al., 1996; Borràs et al., 1999; Attems, 2005; Pekcec et al., 2008). To determine neurological function, we used a series of motor assays. Satisfactory locomotor recovery in TBI canines treated with 3D-CS-bFGF-induced ST was observed not only in the acute stage but also in the chronic stage.

In regard to vivo, bFGF-pretreated stem cell-based therapies for 3D-CS scaffolds represent a promising strategy for brain tissue regeneration through comprehensive regulation of the pathologically hostile microenvironment based on histological analysis. From that, we could observe the orderly arrangement with nerve regeneration in the presence of 3D-CS-bFGF-ST. In addition, we established the simultaneous effect of bFGF-ST in cell-seeded 3D-CS-bFGF-ST on increased cell regeneration by immunofluorescence; positive markers, such as NF, GAP43 and NeuN, were increased, which promoted the formation of nerve fibers, axons and mature neurons and thereby protected against cell death after injury (Choi et al., 2012). In addition, by quantitatively measuring cell proliferation and differentiation during culture in the complexes of the two types, we found that the value of the MMT assay was significantly higher in the 3D-CS-bFGF-ST group than in the 3D-CS-ST group; consistent with our results, these findings suggested that a structurally biomimetic 3D neuronal network *in vivo* was constructed fortunately that led to numerous neurons with functional connections between them. Furthermore, sufficient vascularization remains a major problem, affecting the viability of the scaffold in the long term. A higher level of vWF expression in the injured site was observed in the 3D-CS-bFGF-ST group than in the 3D-CS-ST group.

The balance between anti-inflammatory and proinflammatory cytokines might trigger secondary injury after TBI(Dekmak et al., 2018). Many studies have shown that TNF-α and IL-6 contribute to the pathogenesis and exacerbate traumatic-induced brain damage. Our data suggested that 3D-CS-bFGF-ST could inhibit the expression of TNF-α and IL-6 while increasing IL-10 protein content in the surroundings of the injured area compared with the TBI group. The 3D-CS-bFGF-ST treatment suppressed acute and chronic inflammation in the canine model.

## Conclusion

In this study, the 3D-CS-based scaffold focused on the merits of blending collagen or silk fibroin. Additionally, to our knowledge, there are no published studies, indicating the effect of a combination of collagen/silk fibroin and the secretome derived from HUCMSCs pretreated with bFGF. Our study indicated the safety and efficacy profile of these complexes under manufacturing and quality control. HUCMSC-derived ST pretreated with bFGF as a substitute for cell therapy makes the clinical application of HUCMSCs possible. Implanting 3D-CS-bFGF-ST reduced the cavity area and glial scar, and enhanced the formation of nerve fibers, myelin sheaths, synapses and neurons in the TBI injury area. Furthermore, implanting 3D-CS-bFGF-ST could enhance angiogenesis, reduce cell apoptosis and modulate inflammatory levels after TBI. In future studies, more investigations are needed to provide evidence on the safety and efficacy of 3D-CS-bFGF-ST in the treatment of TBI.

## Data Availability

The original contributions presented in the study are included in the article/supplementary materials, further inquiries can be directed to the corresponding authors.
